# BMP-2-Loaded Self-Crosslinking CaP/Hydrogel Composite Enables Complete Regeneration of Critical-Sized Segmental Bone Defects

**DOI:** 10.3390/bioengineering13080888

**Published:** 2026-07-31

**Authors:** Amadou Touré, Ombeline Aroux, Joelle Veziers, Sophie Sourice, Kevin Minier, Borhane Fellah, Valérie Geoffroy, Bernard Giumelli, Olivier Gauthier, Pierre Weiss

**Affiliations:** 1 UMR 1229, RmeS, Regenerative Medicine and Skeleton, INSERM, CHU Nantes, Oniris, University of Nantes, F-44000 Nantes, France; amadou.toure@ucad.edu.sn (A.T.); e207752s@etu.univ-nantes.fr (O.A.); joelle.veziers@univ-nantes.fr (J.V.); sophie.sourice@oniris-nantes.fr (S.S.); valerie.geoffroy@univ-nantes.fr (V.G.); bernard.giumelli@univ-nantes.fr (B.G.); olivier.gauthier@oniris-nantes.fr (O.G.); 2 Department of Odontology, Faculty of Medicine, Pharmacy and Odontology, University Cheikh Anta DIOP, Dakar BP 5005, Senegal; 3 Center for Preclinical Research and Investigation, Oniris College of Veterinary Medicine Food, Science and Engineering, 44300 Nantes, France; kevin.minier@oncovet.fr (K.M.); fellahborhane@yahoo.fr (B.F.)

**Keywords:** critical sized defect, bone repair, self-crosslinking bone substitute, calcium phosphate hydrogel, recombinant human bone morphogenetic protein-2, dogs

## Abstract

Critical-size segmental bone defects remain a major challenge in orthopedic surgery, often requiring complex reconstruction strategies associated with significant morbidity. This study evaluated the regenerative potential of a self-crosslinking bone substitute (SCBS) composed of biphasic calcium phosphate (BCP) granules suspended in a silanized hydroxypropyl methylcellulose (Si-HPMC) hydrogel, with or without recombinant human bone morphogenetic protein-2 (rhBMP-2), in a canine load-bearing defect model. Bilateral 2-cm segmental defects were created in the ulnae of five adult beagle dogs. Defects were filled with SCBS alone or SCBS loaded with rhBMP-2. Bone regeneration and biomaterial remodeling were assessed after 20 weeks using micro-computed tomography (micro-CT), scanning electron microscopy (SEM), histomorphometry, elemental analysis, and histology. SCBS loaded with rhBMP-2 resulted in complete defect bridging, with 35% newly formed bone and only 5% residual BCP granules. In contrast, SCBS alone induced limited bone formation (10%), primarily at host interfaces, with substantial persistence of BCP (33%). Newly formed bone in the rhBMP-2 group exhibited a dense lamellar structure with Haversian organization and direct contact with residual biomaterial. Elemental analysis revealed a lower Ca/P ratio compared with control, suggesting ongoing remodeling. These findings demonstrate that controlled delivery of rhBMP-2 from a self-crosslinking CaP/hydrogel composite enhances both bone formation and biomaterial resorption, supporting a coupled regeneration process. This approach represents a promising strategy for the treatment of segmental bone defects and non-unions in orthopedic applications.

## 1. Introduction

Critical-size segmental bone defects remain a major challenge in orthopedic surgery, particularly in cases of trauma, tumor resection, or non-union. Current treatments rely on autologous bone grafting, which remains the gold standard but is associated with donor-site morbidity, limited availability, and variable clinical outcomes [1].

Bone tissue engineering approaches aim to overcome these limitations by combining scaffolds, osteogenic cells, and bioactive factors [2]. Among these strategies, biomaterial-based delivery systems for osteoinductive growth factors such as bone morphogenetic protein-2 (BMP-2) have shown significant potential to enhance bone regeneration [1,3].

Biphasic calcium phosphate (BCP) ceramics are widely used as osteoconductive scaffolds due to their biocompatibility and favorable degradation profile [4]. However, when used alone, they are insufficient to regenerate critical-size defects [5]. The addition of BMP-2 has been shown to significantly improve bone formation, particularly in load-bearing models [6,7].

However, the clinical application of BMP-2 remains limited by its short biological half-life, rapid diffusion, and burst release from conventional carriers such as collagen sponges, often leading to the use of supraphysiological doses and associated adverse effects [8,9,10]. This uncontrolled release profile compromises both safety and efficacy, highlighting the need for delivery systems capable of providing sustained and localized growth factor release [10].

Recent advances in biomaterials have emphasized the importance of spatiotemporal control of BMP-2 delivery [10]. In particular, hydrogel-based systems have emerged as promising carriers due to their ability to encapsulate bioactive molecules, prolong their retention at the implantation site, and modulate release kinetics [11]. These systems can improve osteogenic outcomes while reducing systemic exposure and dose-related complications [11].

To address these limitations, we developed a self-crosslinking bone substitute (SCBS) combining BCP granules with a silanized hydroxypropyl methylcellulose (Si-HPMC) hydrogel. This injectable system forms an in situ crosslinked three-dimensional network capable of stabilizing the biomaterial and enabling controlled release of encapsulated growth factors [12,13,14].

A preliminary qualitative evaluation of this approach in a similar canine segmental defect model has been previously reported, demonstrating the feasibility of BMP-2 delivery using a self-crosslinkable CaP/hydrogel construct, although without quantitative or microstructural analyses [15].

Segmental defect models in large animals are considered highly relevant for evaluating bone regeneration under load-bearing conditions and provide strong translational value for clinical applications [16].

The aim of this study was to investigate the capacity of rhBMP-2 delivered from a self-crosslinking CaP/hydrogel composite to promote bone regeneration, biomaterial remodeling, and mineral maturation in a canine critical-size segmental defect.

## 2. Materials and Methods

### 2.1. Self-Crosslinking Bone Substitute

The self-crosslinking bone substitute (SCBS) consisted of biphasic calcium phosphate (BCP) granules suspended in a silanized hydroxypropyl methylcellulose (Si-HPMC) hydrogel. The BCP granules (0.5–1.0 mm; Graftys, Aix-en-Provence, France) were macro- and microporous calcium phosphate ceramics. The hydroxyapatite/β-tricalcium phosphate ratio was 60/40 by weight, as determined by energy-dispersive X-ray spectroscopy (EDX).

The hydrogel component was based on silanized hydroxypropyl methylcellulose (Si-HPMC), previously developed for injectable tissue engineering applications. Si-HPMC powder was dissolved in 0.2 M NaOH at 25 °C for 48 h under constant stirring. The resulting solution was dialyzed in 0.09 M NaOH and then aliquoted and sterilized by autoclaving at 121 °C for 30 min.

Crosslinking was induced by adding an acidic HEPES-based buffer (pH 3.6) to the Si-HPMC solution at a 2:1 volume ratio, yielding a self-crosslinking hydrogel at physiological pH.

### 2.2. rhBMP-2 Preparation

The osteoinductive factor used was recombinant human BMP-2 (rhBMP-2; Dibotermin alfa; TruScient kit, Pfizer, New York, NY, USA). The lyophilized product was reconstituted using the liquid Pfizer kit according to a modified protocol to obtain a final rhBMP-2 concentration of 0.2 mg/mL within the hydrogel.

### 2.3. Preparation of the Constructs

For the control condition, 1 mL of Si-HPMC hydrogel was mixed with 600 mg of BCP granules without rhBMP-2.

For the experimental condition, rhBMP-2 was incorporated into the Si-HPMC hydrogel immediately before implantation. Briefly, 0.25 mL of the rhBMP-2 solution was mixed with 0.25 mL of acidic buffer and then combined with 0.5 mL of Si-HPMC (4%), yielding 1 mL of hydrogel containing rhBMP-2 at 0.2 mg/mL. BCP granules (600 mg) were then suspended in the hydrogel to obtain a moldable composite in a 50/50 (*w*/*v*) ratio.

### 2.4. Animals

All procedures were conducted in accordance with European guidelines for animal care and use and were approved by the Pays de la Loire Animal Ethics Committee (CEEA Pays de la Loire No. 2012.120) and the local animal welfare committee of ONIRIS, Nantes, France.

Five healthy adult female beagle dogs that were 8 years old were included in the study.

### 2.5. Surgical Procedure

Premedication consisted of morphine administered 30 min before induction of general anesthesia. Anesthesia was induced with propofol (6 mg/kg IV) and maintained with isoflurane in 100% oxygen. Cefalexin (30 mg/kg IV) was administered perioperatively for antibiotic prophylaxis.

With the animals in lateral recumbency, the mid-diaphysis of each ulna was exposed through a standard surgical approach. A 2-cm critical-size segmental defect was created in each ulna using an oscillating saw under continuous saline irrigation, including resection of the periosteum. The ulna was stabilized using an 8-hole 2.4-mm locking compression plate (eight-hole LCP^®^ 2.4 mm Depuy-Synthes, Raynham, MA, USA) fixed with five screws.

The left defects were filled with SCBS loaded with rhBMP-2, whereas the right defects received SCBS without rhBMP-2. Wounds were closed in layers with watertight sutures.

Postoperative care included meloxicam (0.1 mg/kg orally for 5 days) and daily local wound care for 3 days.

### 2.6. Micro-CT Analysis

Animals were euthanized 20 weeks after implantation. Explants were harvested with 2 cm of bone on either side of the defect while preserving the proximal and distal host bone interfaces.

Samples were analyzed using a Skyscan-1272 high-resolution 3D X-ray micro-computed tomography (micro-CT) system Bruker (Kontich, Belgium). The scanner was equipped with a 40 to 100 kV (10 W) X-ray source and an 11-megapixel X-ray detector. Each sample was placed on a holder and scanned using a 0.25 mm aluminum filter, at 70 kV–142 µA, with the following parameters: resolution: 7 µm isotropic voxels; rotation step: 0.45° on 360°; and frame averaging: 2.

For 3D reconstruction we used NRecon software (1.6.9.8, Bruker^®^) with the following parameters: smoothing, ring artifact correction, and beam hardening correction, set to 2, 14, and 14% respectively.

The 3D reconstructions of the samples were subsequently used for quantitative analysis. The reconstructed µCT datasets were segmented using Dragonfly software (version 2025.1.0.2063 Comet Technologies, Quebec, Canada). Analysis was performed using Dragonfly software based on a deep learning approach. Regions of interest (ROIs) were defined using a cube that was subsequently stretched to obtain a rectangular parallelepiped (cuboid), with a fixed dimension of 20,000 µm along the Z-axis and X- and Y-axis dimensions determined by the surface area of the bone and biphasic calcium phosphate (BCP).

3D Analysis: First, an initial model was generated from the images corresponding to the ROI. This step involved segmenting several regions on different image slices. For each region, the pixels corresponding to the different components (bone, BCP, and empty space/soft tissue) were manually labeled by assigning specific colors. Subsequently, a training step was performed during which the software was trained using a deep learning process.

Once the first version of the model had been generated, it was applied to the ROI and the results were evaluated. Any segmentation errors were corrected by performing additional segmentations following the same procedure described above. During these correction steps, the deep learning algorithm provided predictions for color assignment, which could subsequently be manually adjusted if necessary. This iterative process of correction and model refinement was repeated progressively to improve the model until satisfactory segmentation results were obtained.

### 2.7. SEM and EDX Analysis

For each animal model (8-year-old female beagle), four (4) samples were analyzed by SEM and EDX, two with BMP (left ulna) and two without BMP (right ulna). The explants were inserted in a medio-distal (cranio-caudal) direction; each block was sectioned longitudinally in the middle (yielding two samples). This included the upper, middle, and lower layers.

For SEM analysis, samples were polished, sputter-coated with gold–palladium, and examined in backscattered electron mode using a Leo 1450VP microscope (Zeiss, Oberkochen, Germany). Quantitative 2D histomorphometric analysis was performed using Quantimet 500 software (Leica, London, UK). The following parameters were measured within the defect area: bone surface/total surface (BS/TS), residual biomaterial surface/total surface (BiomatS/TS), and soft tissue surface/total surface (Soft tissue S/TS).

Elemental analysis was performed by EDX (Link Isis, Oxford Instruments, Oxfordshire, UK). The calcium/phosphorus (Ca/P) ratio was measured in newly formed bone and compared between groups and with adjacent native bone. Microanalysis was performed using energy-dispersive X-ray spectroscopy coupled to scanning electron microscopy with an accelerating voltage of 100 kV. The calcium/phosphorus (Ca/P) ratio was compared in the newly formed bone observed in samples treated or not with BMP-2 after explantation, as well as with the Ca/P ratio of the adjacent native bone. The Ca/P ratio is expressed as mean ± standard deviation obtained from 16 measurements on each sample.

### 2.8. Histology

Non-decalcified specimens were embedded in glycol methacrylate resin. Sections 5 µm thick were cut parallel to the long axis of the ulna and stained with hematoxylin and eosin. Slides were examined by light microscopy. Five slides were analyzed for each sample.

### 2.9. Statistical Analysis

Data are presented as mean ± standard deviation. Groups were compared using analysis of variance (ANOVA) followed by Fisher’s PLSD post hoc test. Statistical significance was set at *p* < 0.05.

## 3. Results

### 3.1. Clinical Outcomes

Postoperative recovery was uneventful in all animals. No wound dehiscence, swelling, or infection was observed. All dogs resumed full weight-bearing within 48 h after surgery. The osteosynthesis plate (eight-hole LCP^®^ 2.4 mm Depuy-Synthes (Locking Compression Plate)) was always attached after 20 weeks.

### 3.2. Micro-CT Analysis

**Figure 1:**
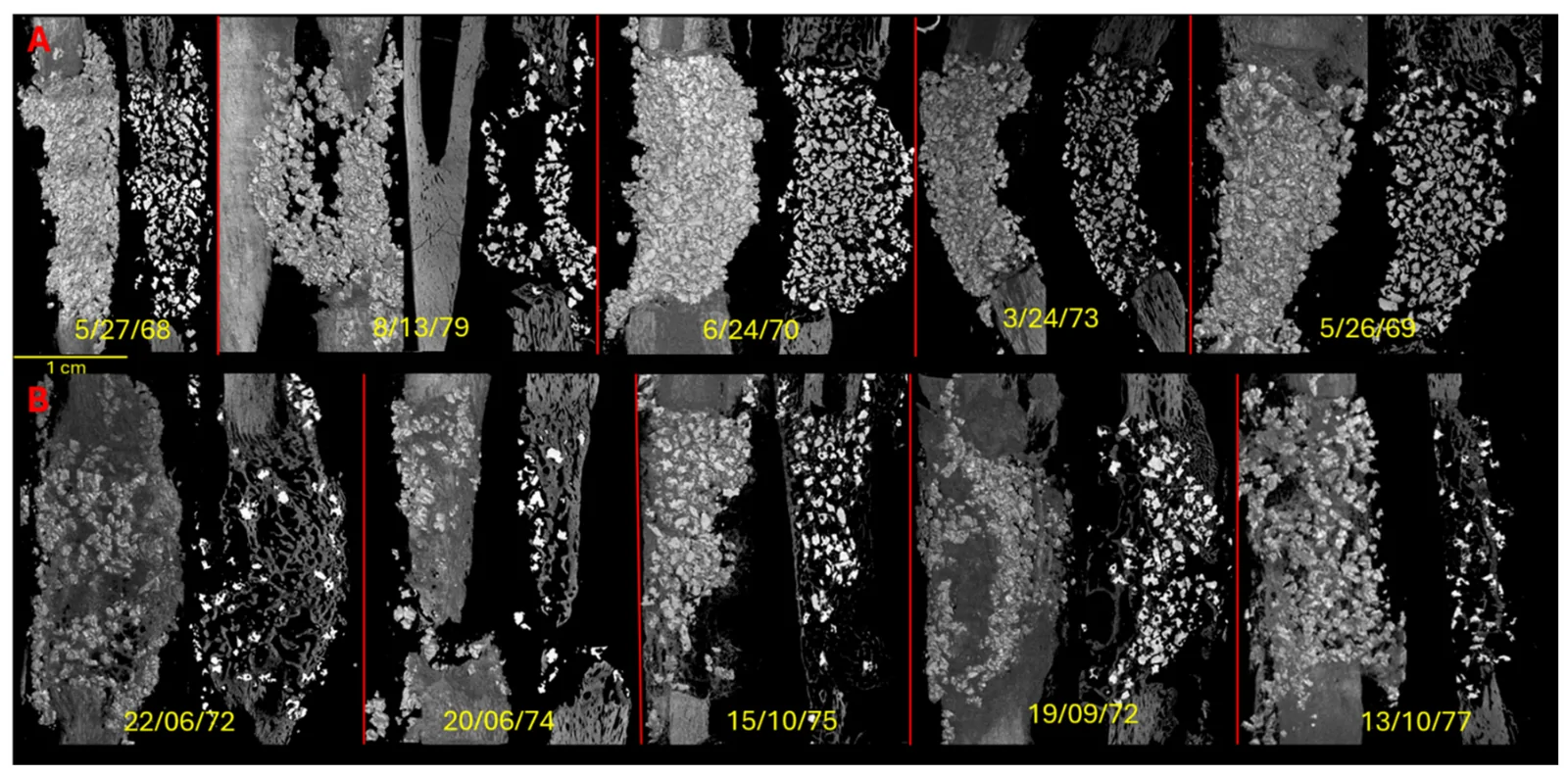
Three-dimensional micro-CT reconstructions of canine ulnar defects 20 weeks after implantation and a 2D representation in the centrum of the reconstruction. Scans were acquired at 100 kV and 91 µA with a pixel size of 19.4 µm. Defects were filled with: (**A**) SCBS without rhBMP-2; (**B**) SCBS loaded with rhBMP-2. Scale bar represent 1 cm. In yellow, % of 3D measurements using deep learning dragonfly software: (New Bone/Biomaterial/Soft tissue).

At 20 weeks, micro-CT showed marked differences between the two groups (Figure 1). In the SCBS-only group, the composite remained in place without evidence of leakage, but the defect was largely occupied by persistent BCP granules surrounded by poorly mineralized tissue. Limited bone formation was observed only at the interfaces with the host bone.

In contrast, defects treated with SCBS loaded with rhBMP-2 showed complete bridging by dense newly formed bone for 4 dogs. The regenerated tissue exhibited a three-dimensional architecture similar to native bone and was intimately associated with the residual BCP granules. Residual ceramic volume was markedly reduced in this group, suggesting active biomaterial remodeling.

### 3.3. SEM and Histomorphometry

**Figure 2:**
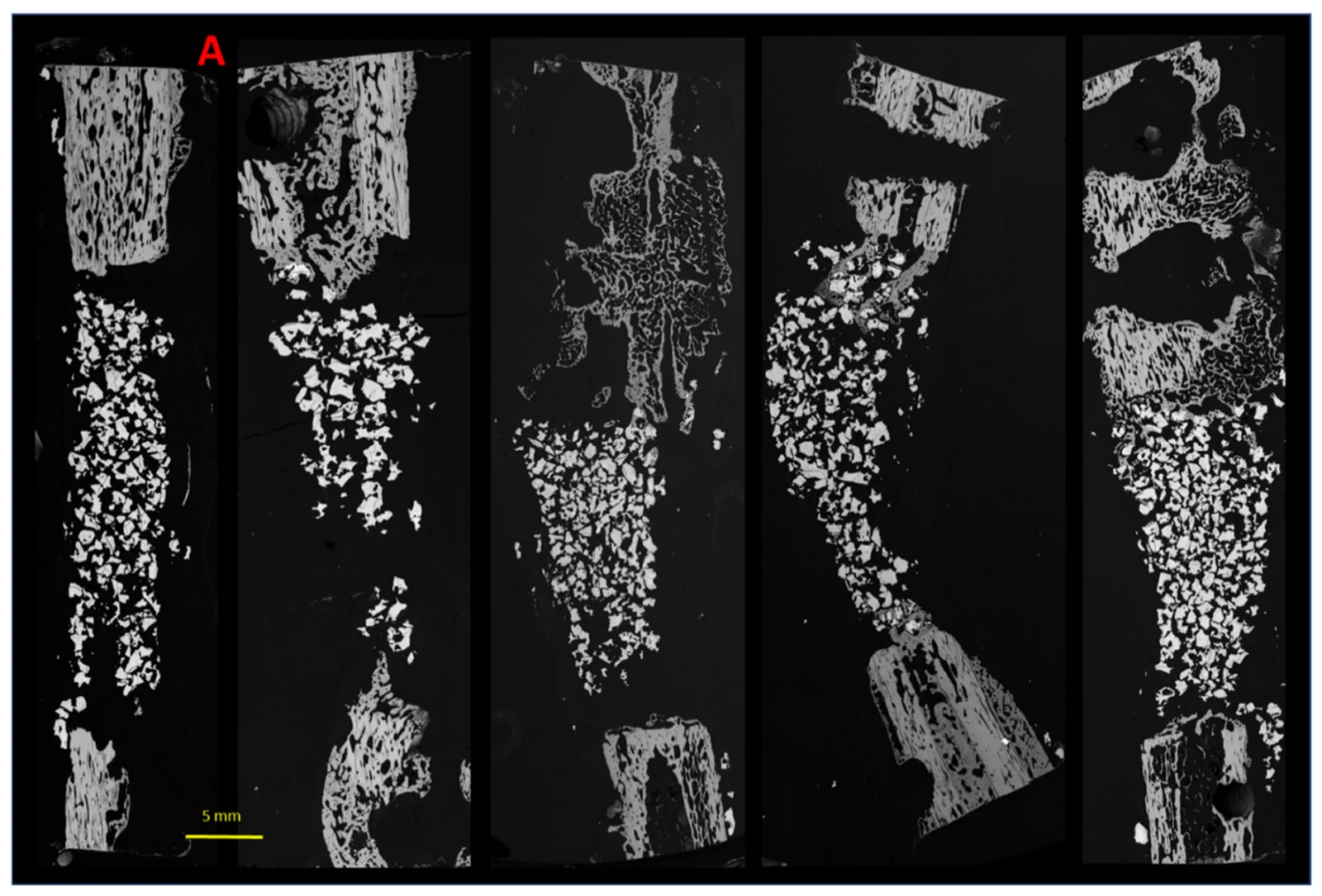

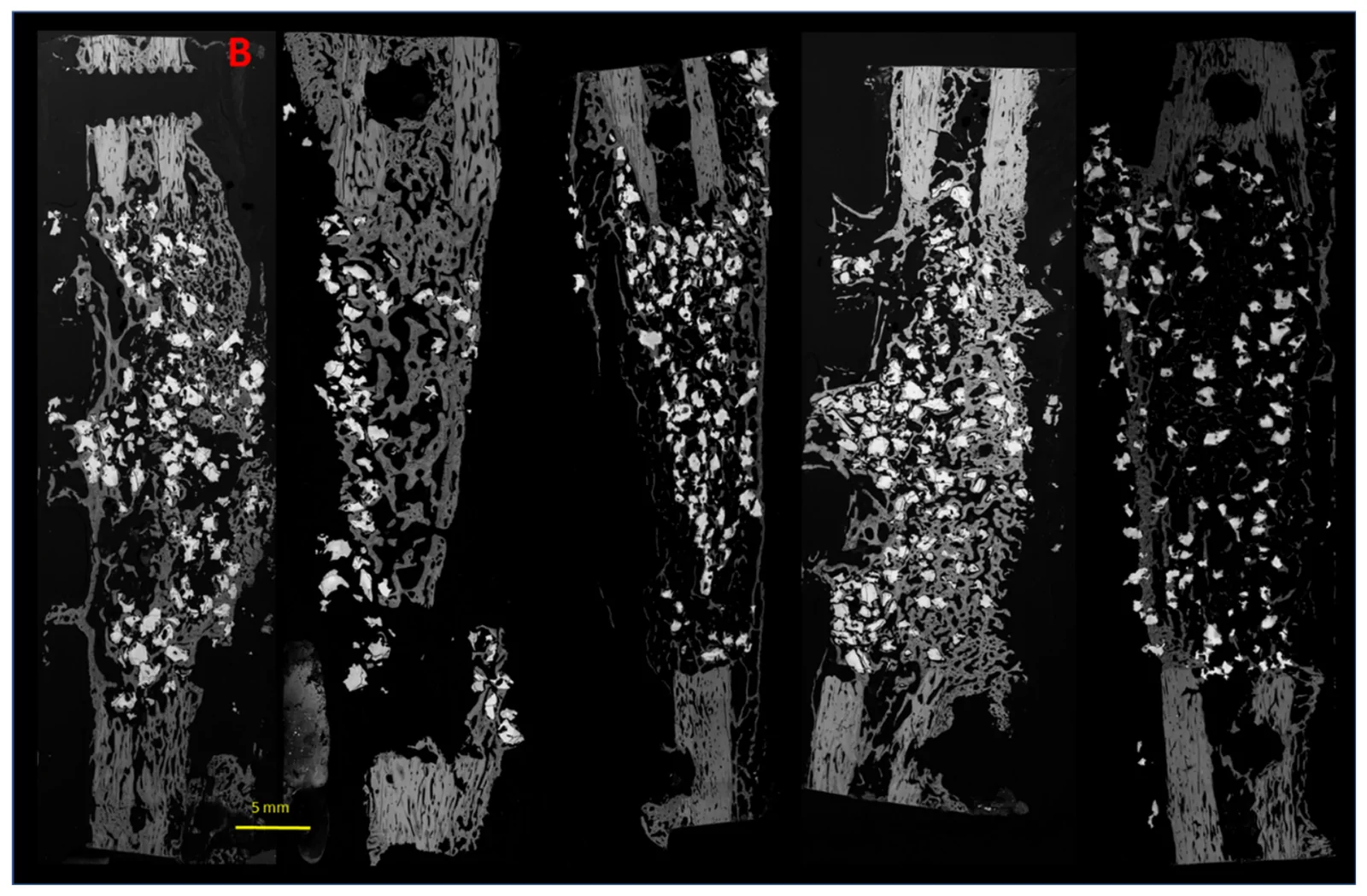
Representative backscattered SEM images of canine ulnar defects 20 weeks after implantation. (**A**) SCBS without rhBMP-2; (**B**) SCBS loaded with rhBMP-2. In grayscale images, bone appears gray, biomaterial white, and soft tissue black.

SEM confirmed the micro-CT findings (Figure 2). In the absence of rhBMP-2, only limited osteoconduction was observed at the proximal and distal interfaces, with no substantial bone formation in the center of the defect and persistence of a large amount of BCP granules.

By contrast, SCBS loaded with rhBMP-2 induced abundant lamellar bone formation throughout the entire defect. Newly formed bone was in direct contact with both host bone and BCP granules and showed a Haversian-like organization with numerous osteocyte lacunae.

**Figure 3:**
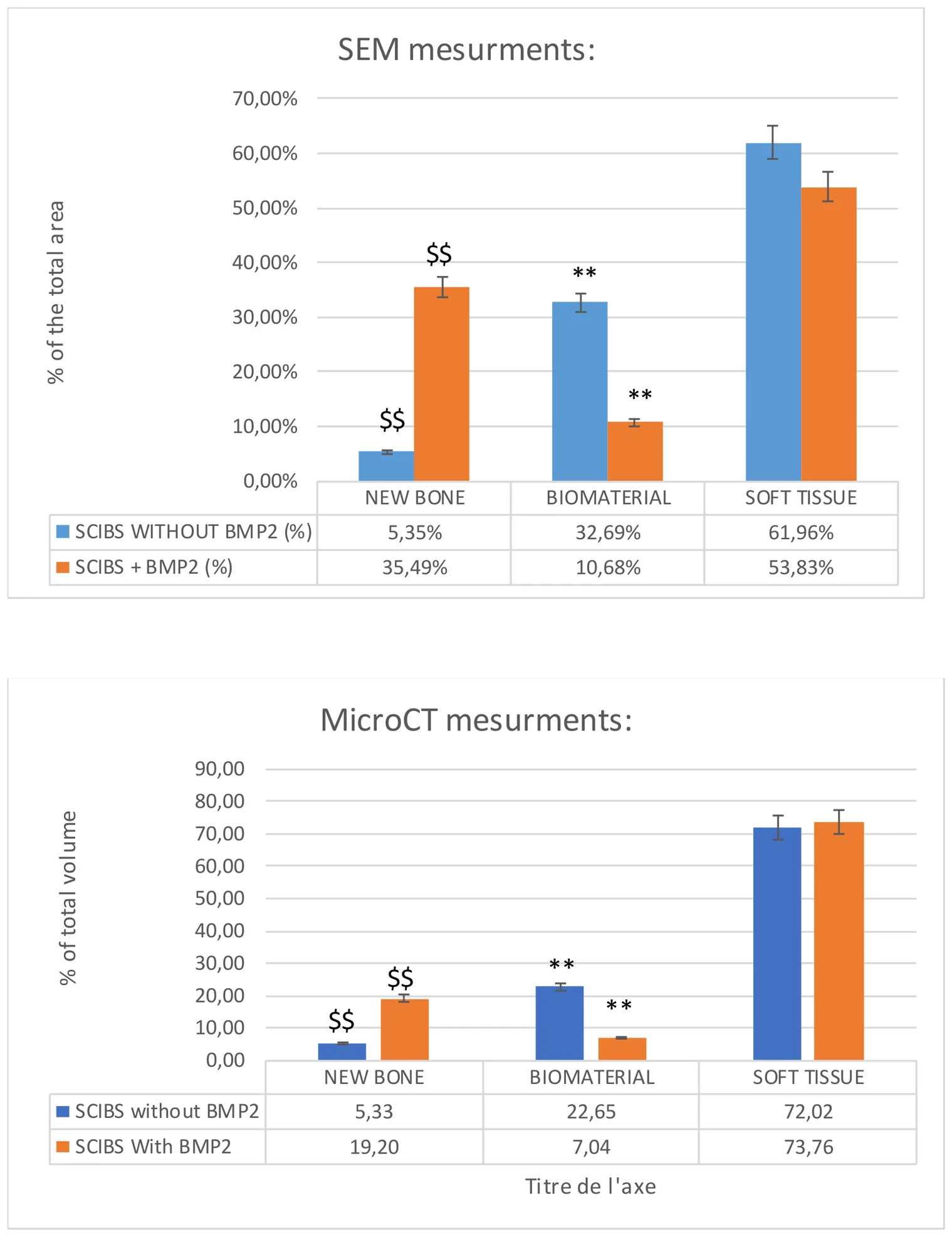
Quantitative SEM and micro CT (using Dragonfly software deep learning) histomorphometry of defect composition. Bone surface fraction was significantly higher in defects treated with rhBMP-2, whereas residual BCP surface fraction was significantly lower. *p* < 0.0001.

Quantitative SEM analysis (Figure 3) demonstrated significantly greater bone formation in the rhBMP-2 group than in the control group (35% vs. 10%, *p* < 0.0001). Conversely, the percentage of residual BCP granules was significantly lower in the rhBMP-2 group (5% vs. 33%, *p* < 0.0001).

Using micro-CT dragonfly software deep learning analyses, the results are quantitively different with 5% of new bone and 22% of BCP granules using SCIBS without BMP-2. In the SCIBS with BMP-2 conditions, analyses show 20% of new bone and 10% of BCP granules with BMP-2.

### 3.4. Elemental Analysis

EDX analysis (Figure S1 and Table 1) confirmed the mineralized nature of the newly formed tissue. Bone formed in the rhBMP-2 group showed calcium and phosphorus levels closer to those of native bone than bone formed in the control group. However, the Ca/P ratio remained lower in the rhBMP-2 group (1.54) than in the control group (1.86), suggesting that active mineral maturation and remodeling were still ongoing at 20 weeks.

### 3.5. Histology

**Figure 4:**
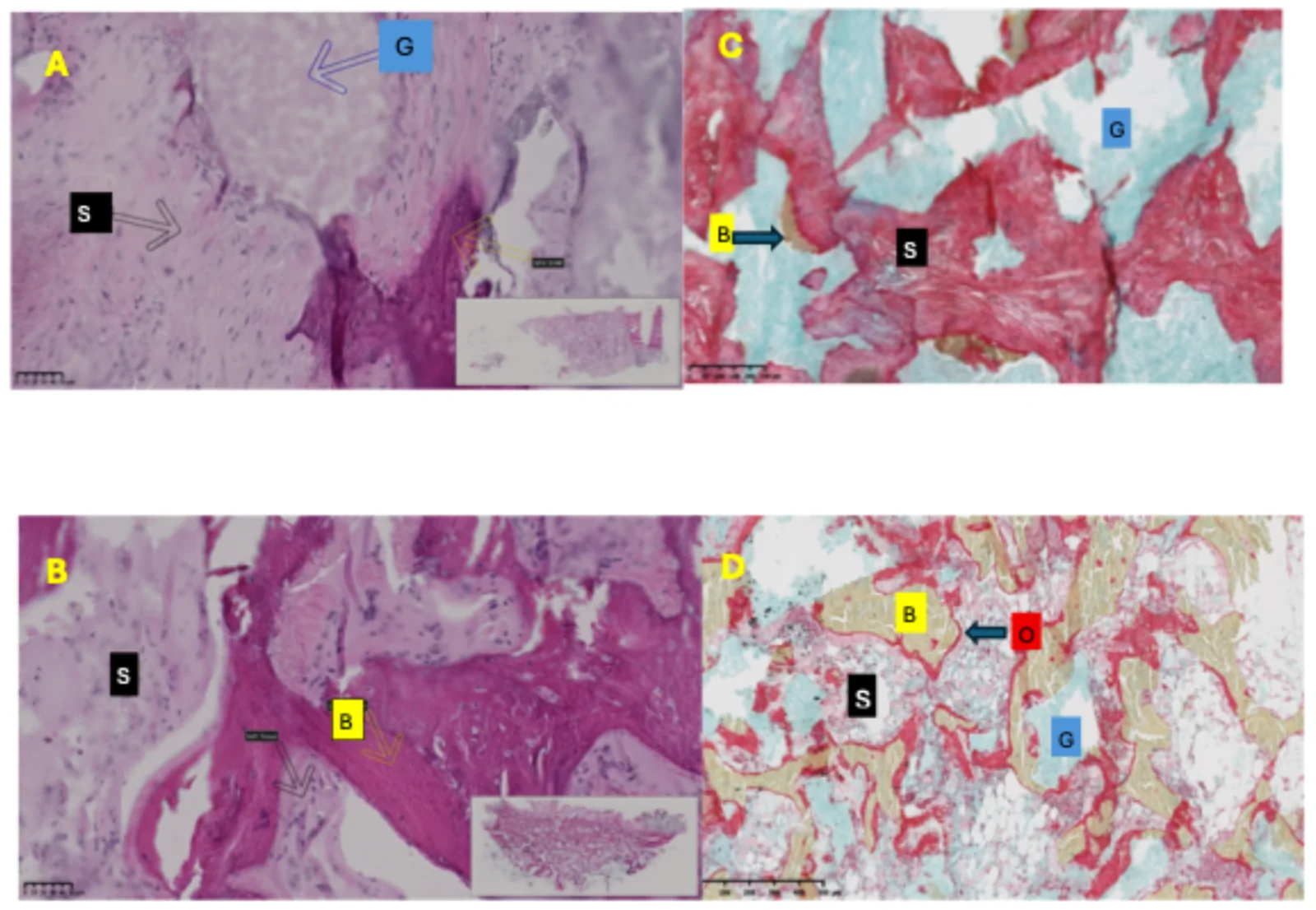
Histological evaluation of bone formation: hematoxylin-eosin-stained sections 20 weeks after implantation (**A**,**B**) (Scale bar: 50 µm). Newly formed bone is indicated by yellow square B, residual BCP granules by blue square G, and soft tissue by black square S. Scale bar = 50 µm. MOVAT pentachrome staining (**C**,**D**) (Scale bar: 250 µm) marked the biomaterial in blue G, the new bone in green B, the osteoid barrier in dark red O, and soft tissue in red S. (**A**–**C**) SCBS without rhBMP-2; (**B**–**D**) SCBS loaded with rhBMP-2.

Histological observations (Figure 4) were consistent with the imaging data. In the control group, BCP granules were embedded in fibrovascular connective tissue, with only limited new bone formation at the defect margins.

In contrast, defects treated with SCBS plus rhBMP-2 were completely filled with dense lamellar bone containing numerous osteocytes. Trabeculae were continuous with the host cortical bone and periosteum, and newly formed vessels were visible within the regenerated tissue. Multinucleated cells adjacent to residual granules suggested active biomaterial resorption and remodeling. MOVAT pentachrome staining shows a high level of osteoid that seems to highlight the mineralization process with SCBS plus rhBMP-2.

## 4. Discussion

This study demonstrates that controlled delivery of rhBMP-2 from a self-crosslinking CaP/hydrogel composite significantly enhances bone regeneration in a critical-size segmental defect. While the SCBS alone exhibited limited osteoconductive properties, it was insufficient to achieve complete defect healing. In contrast, rhBMP-2 delivery resulted in complete bridging and the formation of dense, lamellar bone with Haversian organization.

The newly formed bone exhibited structural and compositional features consistent with mature bone tissue, including osteocyte-rich architecture and vascularization [17,18]. These findings indicate effective osteointegration and functional regeneration rather than simple mineral deposition. A limited amount of ectopic bone formation was observed in some samples. This may not solely reflect uncontrolled BMP-2 diffusion but could also result from slight displacement of the CaP/Si-HPMC composite after implantation. Because the hydrogel remains relatively compliant after crosslinking, postoperative micromotions may induce migration of composite fragments containing both BMP-2 and BCP granules, thereby promoting localized bone formation outside the initial defect boundaries. We performed ELISA evaluation of BMP-2 in the supernatant after day 3 of SCBS in different solvents at 1/10 and 1/500 dilutions. BMP-2 ELISA assay at day 3 at 1/10 and 1/500 dilutions in different solvents showed that almost no rapid release was observed (Figure S2).

A key observation was the significant reduction in residual BCP in the rhBMP-2 group, suggesting an active coupling between biomaterial resorption and bone formation [19,20]. This coupling is essential for successful bone regeneration, as scaffold degradation should ideally match the rate of new tissue formation.

Quantitative results using SEM or Dragonfly micro CT deep learning analysis did not showed the same results with 10% differences because we compared 2D versus 3D and the analysis processing (segmentation) was also different. Both methods show significative differences between biomaterial composites without or with rhBMP2.

The lower Ca/P ratio observed in the rhBMP-2 group suggests that bone remodeling was still ongoing at 20 weeks. This is consistent with previous studies indicating that BMP-2-induced bone formation initially results in immature bone that progressively remodels into lamellar bone [21].

From a mechanistic perspective, the Si-HPMC hydrogel likely plays a critical role in controlling BMP-2 release kinetics, thereby avoiding the burst release associated with collagen carriers [8]. Recent studies have demonstrated that controlled and sustained delivery of BMP-2 significantly improves osteogenic efficiency while reducing adverse effects [10]. Hydrogel-based systems, in particular, allow modulation of growth factor diffusion, retention, and bioavailability at the defect site [11].

The present study significantly extends our previous qualitative observations in a comparable canine segmental defect model [13]. While the earlier work demonstrated the feasibility of BMP-2 delivery from a self-crosslinkable CaP/hydrogel construct, it did not provide quantitative or mechanistic insights. In contrast, the current study offers a comprehensive evaluation combining micro-CT, SEM-based histomorphometry, and compositional analyses.

Importantly, these findings highlight the dual role of BMP-2 not only in promoting robust bone formation but also in accelerating calcium phosphate resorption, suggesting a coupled remodeling process. These results also highlight that the BMP-2 also allows the hydrogel degradation because crosslinked HPMC acts like a barrier for bone substitution in a rabbit model [22]. This synergy between osteoinduction and biomaterial degradation represents a key mechanism for achieving functional bone regeneration in critical-sized defects.

These findings are consistent with recent advances in orthopedic biomaterials, where composite scaffolds combining calcium phosphates and growth factors are increasingly investigated as alternatives to autografts [23]. Calcium phosphate-based materials not only provide osteoconductive support but also act as reservoirs for growth factor delivery, enhancing local osteoinduction [24].

Clinically, these results are highly relevant for orthopedic applications, particularly in the treatment of segmental defects, delayed unions, and non-unions [2]. The ability of this composite system to promote complete defect bridging in a load-bearing model highlights its strong translational potential.

Moreover, the injectable and moldable properties of the hydrogel-based composite may facilitate minimally invasive surgical approaches and improve adaptation to irregular defect geometries, which is a key advantage over preformed scaffolds.

Future developments in bone tissue engineering increasingly rely on multifunctional scaffolds integrating growth factor delivery, biomaterial optimization, and vascularization strategies to enhance clinical translation [25]. In this context, the present composite system represents a promising platform for next-generation bone substitutes.

## 5. Conclusions

The present study demonstrates that a self-crosslinking CaP/hydrogel composite delivering rhBMP-2 can effectively regenerate critical-size segmental bone defects, promoting both bone formation and biomaterial resorption. This study provides the first in vivo quantitative and microstructural characterization of a BMP-2 delivery self-crosslinkable CaP/hydrogel construct in a clinically relevant large animal model.

This approach enables a coupled regeneration process and represents a promising strategy for the treatment of large bone defects and non-unions in orthopedic surgery.

## Figures and Tables

**Table 1:** Comparison of Ca/P ratios in newly formed bone and native bone.

	Newly Formed Bone SCBS Without BMP	Newly Formed Bone SCBS + BMP	Host Bone
Calcium Ca	15.87 (±2.33)	20.03 (±1.88)	32.77 (±0.16)
Phosphorus P	8.55 (±0.88)	12.98 (±0.77)	15.92 (±0.16)
Ratio Ca/P	**1.86**	**1.54**	**2.06**

## Data Availability

The original contributions presented in this study are included in the article/Supplementary Materials. Further inquiries can be directed to the corresponding author.

## Supplementary Materials

The following supporting information can be downloaded at: https://www.mdpi.com/article/10.3390/bioengineering13080888/s1, Figure S1: Backscattered high resolution SEM images with 1 exemple of area of EDX analysis (Spectre1). Exemple of A: EDX Spectra of host bone. B: EDX Spectra of New Bone. Figure S2: BMP-2 ELISA assay of the supernatant after day 3 of SCBS in different solvents at 1/10 and 1/500 dilutions.
