# Supplementary material for: BMP-2-Loaded Self-Crosslinking CaP/Hydrogel Composite Enables Complete Regeneration of Critical-Sized Segmental Bone Defects
Source: Bioengineering (Basel). 2026 Jul 31;13(8):888. doi: 10.3390/bioengineering13080888 (PMC13509907; doi:10.3390/bioengineering13080888)
Supplement: Supplementary file 1 [file bioengineering-13-00888-s001.zip › bioengineering-4347694-supplementary.pdf]

SUPLEMENTARY DATA :

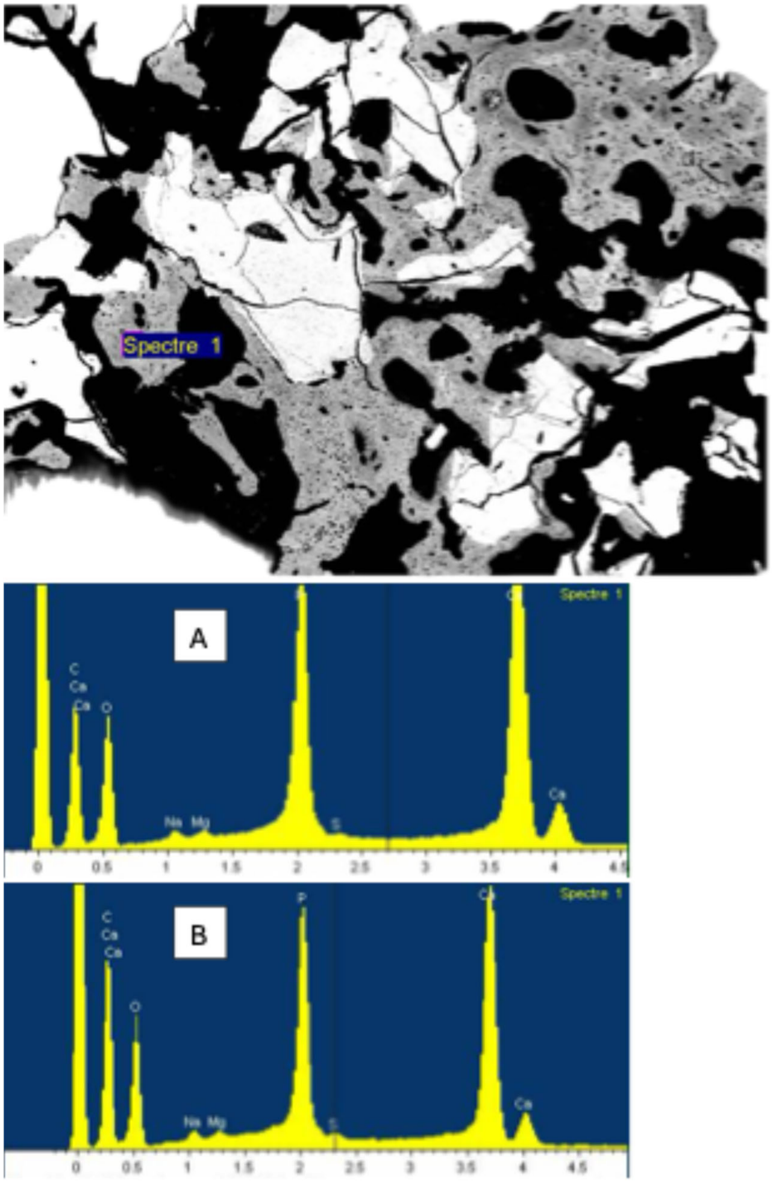

Figure S1 : Backscattered high resolution SEM images with 1 exemple of area of EDX analysis (Spectre1). Exemple of A: EDX Spectra of host bone. B: EDX Spectra of New Bone

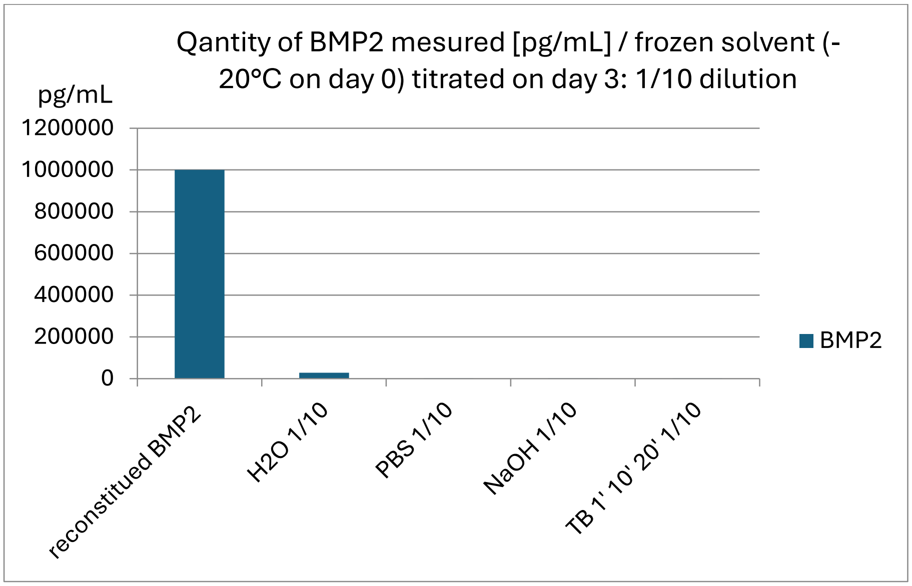

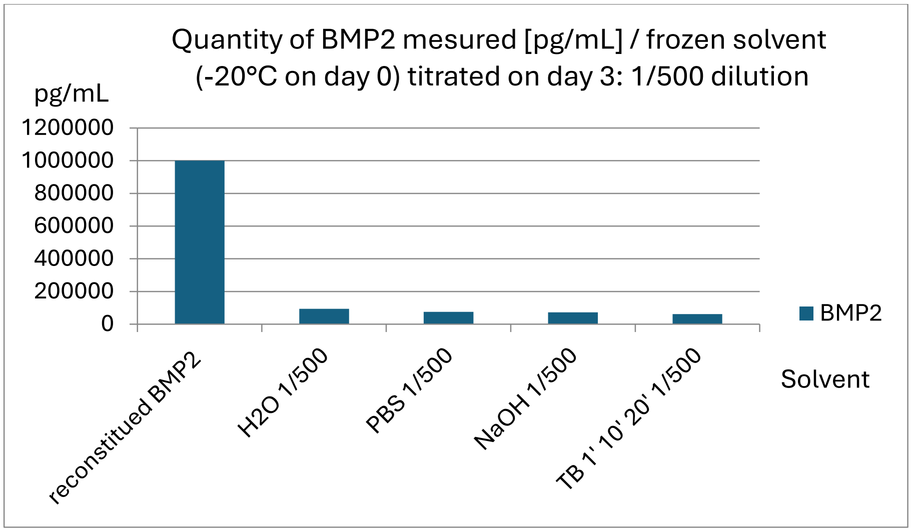

Figure S2 : BMP2 ELISA assay of the supernatant after day 3 of SCBS in different solvents at 1/10 and 1/500 dilutions.
